# Machine Learning Prediction of Analyte-Induced Fluorescence Perturbations in DNA-Functionalized Carbon Nanotubes

**DOI:** 10.1021/acs.nanolett.5c05206

**Published:** 2026-01-07

**Authors:** Sayantani Chakraborty, Andrew T. Krasley, Colby H. Smith, Abraham G. Beyene, Lela Vuković

**Affiliations:** † Department of Chemistry and Biochemistry, The University of Texas at El Paso, El Paso, Texas 79968, United States; ‡ Janelia Research Campus, 2405 Howard Hughes Medical Institute, Ashburn, Virginia 20147, United States; § Computational Science Program, The University of Texas at El Paso, El Paso, Texas 79968, United States; ∥ Bioinformatics Program, The University of Texas at El Paso, El Paso, Texas 79968, United States

**Keywords:** single-walled carbon nanotubes, DNA−nanotube conjugates, optical biosensors, dopamine, machine learning, analyte structure−nanosensor response relationship

## Abstract

Single-walled carbon nanotubes (SWCNTs) functionalized with single-stranded DNAs can function as near-infrared nanosensors for molecular analytes. However, predicting which analytes elicit strong optical responses for specific nanosensors remains challenging. We developed machine learning (ML) models to predict analyte-induced fluorescence changes in a DNA–SWCNT dopamine nanosensor. Using a data set of 63 small molecules sampling chemical space around dopamine, we encoded analytes with RDKit fingerprints, with or without HOMO and LUMO energies, and applied principal component analysis to identify structural motifs associated with optical response strength. We trained support vector regression and classification models using two strategies: ensembles of 200 models and cross-validation. Regression models achieved mean *R* ^2^ values of 0.2–0.4, with cross-validation outperforming ensembles, while classifiers reached mean F1 scores of ∼0.8. Cross-validation performed best for predictions on a blind set of 21 molecules. These findings show that ML can capture structure–response patterns in modest data sets and guide in silico DNA–SWCNT nanosensor design.

## Graphical abstract

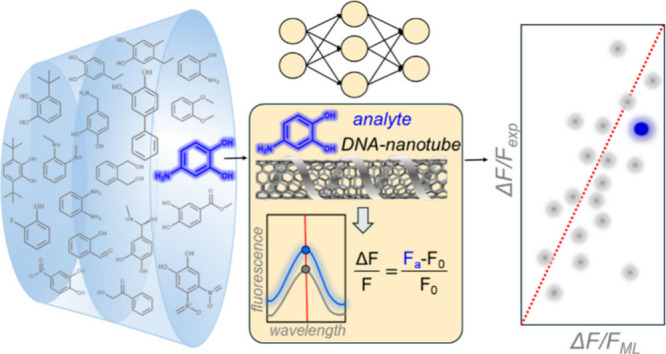

Single-walled carbon nanotubes (SWCNTs) are unique nanomaterials with remarkable optical, electronic, and mechanical properties, enabling applications in biosensing, molecular imaging, drug delivery, and nanoelectronics.^1–11^ A critical step in harnessing these properties is functionalization, often achieved by noncovalent adsorption of polymers that form a corona phase around the nanotube. Such coatings solubilize and stabilize SWCNTs in aqueous environments, without impeding SWCNTs’ inherent near-infrared fluorescence, rendering them biocompatible and useful as reagents for biosensing. Among functionalization strategies, wrapping with single-stranded DNA (ssDNA) oligonucleotides has found widespread use, producing sensors with sequence-dependent specificity toward small molecules,^12–14^ nucleic acids,^15^ and proteins.^16–18^ The optical response of ssDNA-wrapped SWCNTs is dictated by the interplay between the ssDNA sequence and target analyte.^6,19–21^ Despite their potential, identifying ssDNA sequences that simultaneously enable analyte recognition and elicit strong optical signals remains a bottleneck. Typical approaches rely on low-throughput experimental screening of ssDNA libraries, which have occasionally led to serendipitous discoveries of responsive sequences for analytes such as dopamine and norepinephrine.^22,23^ More systematic strategies such as SELEC (systematic evolution of ligands by exponential enrichment on carbon nanotubes) have expanded sequence discovery,^24,25^ but experimental screening of ssDNA–SWCNT responses remains constrained to modest throughput (tens to hundreds of conditions).^12,26,27^

Recent studies have explored the use of machine learning (ML) models trained on small ssDNA–SWCNT data sets to predict ssDNA sequence motifs that recognize specific analytes.^12,26,28,29^ Such models have guided in silico sequence selection for analytes such as serotonin, reducing experimental effort while achieving success rates of 60–90% when predicting new DNA–SWCNT sensors with strong and weak responses to serotonin.^12,26^ These advances suggest that even modest data sets contain chemically meaningful signals that ML can exploit. However, whether ML can predict analyte-induced responses for a given ssDNA–SWCNT conjugate remains largely unexplored. Such a capability would help identify responsive analytes or potential interferents in complex biological environments.

In prior work, we constructed a data set of optical responses of (GT)_6_–SWCNT dopamine nanosensors screened against 63 small molecules spanning the chemical space around dopamine (Figure S1).^30^ This data set provides a foundation to investigate whether analyte structure alone is sufficient to predict fluorescence modulation. Here, we analyzed the data set using principal component analysis (PCA) to identify structural motifs linked to response strength and developed support vector regression (SVR) and support vector classification (SVC) models trained on molecular fingerprints and orbital descriptors. We compared ensemble and cross-validation approaches, evaluated predictive performance, and tested model generalizability using a blind set of 21 molecules. Recent studies have demonstrated the power of high-throughput DNA library screening and multisensor arrays for optimizing detection of specific targets through broad sequence exploration and signal diversity. These methods excel at identifying high-performance sensors for defined analytes and yield robust classifiers for clinical or diagnostic applications.^31–34^ In contrast, our single-sensor, multianalyte framework focuses on sensor selectivity by modeling responses across a structurally diverse chemical space using a single, fixed DNA–SWCNT nanosensor. By emphasizing analyte-driven prediction, our study complements previously reported sequence-focused ML efforts and demonstrates how predictive modeling can inform nanosensor design.

Our data set comprised 63 small molecules tested for Δ*F*/*F*_exp_ responses with (GT)_6_–SWCNT dopamine nanosensors (Figure S1 and Table S1). Δ*F*/*F*_exp_ is defined as the change in fluorescence intensity from baseline (Δ*F*/*F*_exp_ = (*F* – *F*_0_)/*F*_0_, where *F*_0_ and *F* are areas under the fluorescence intensity curves in the wavelength range from 1100 to 1400 nm before and after analyte addition, respectively). The data set was generated using (GT)_6_–SWCNT nanosensors prepared from mixed-chirality SWCNT samples, as described in the extended methods in the Supporting Information; example fluorescence spectra of the nanosensor solution before and after the addition of dopamine analyte are reported in Figure S2. To evaluate whether the analyte structure correlates with the response magnitude, we performed PCA using three molecular encoding methods: extended connectivity fingerprint (ECFP4) bit vectors (radius 2, 2048 dimensions),^35^ ECFP4 count vectors, and molecular access system (MACCS) keys^36^ (Figure 1a–c). Across all representations, molecules producing strong optical responses could be separated from those producing weak responses in the principal component space. For ECFP4 bit vectors, PC1 values correlated strongly with Δ*F*/*F*_exp_ (ρ_PC1_ = 0.602, and *p* < 0.0001; ρ refers to the Spearman coefficient here and below), while PC2 correlated with weakly responding analytes (ρ_PC2_ = −0.221, and *p* = 0.08). Similarly, PC1 values for ECFP4 count vectors correlated with Δ*F*/*F*_exp_ (ρ_PC1_ = 0.649, and *p* < 0.0001). MACCS keys also differentiated strong-response molecules, clustering them in the top left region of PCA space (Figure 1c). These consistent separations across encoding methods confirm that analyte structure encodes information predictive of the sensor response.

**Figure 1:**
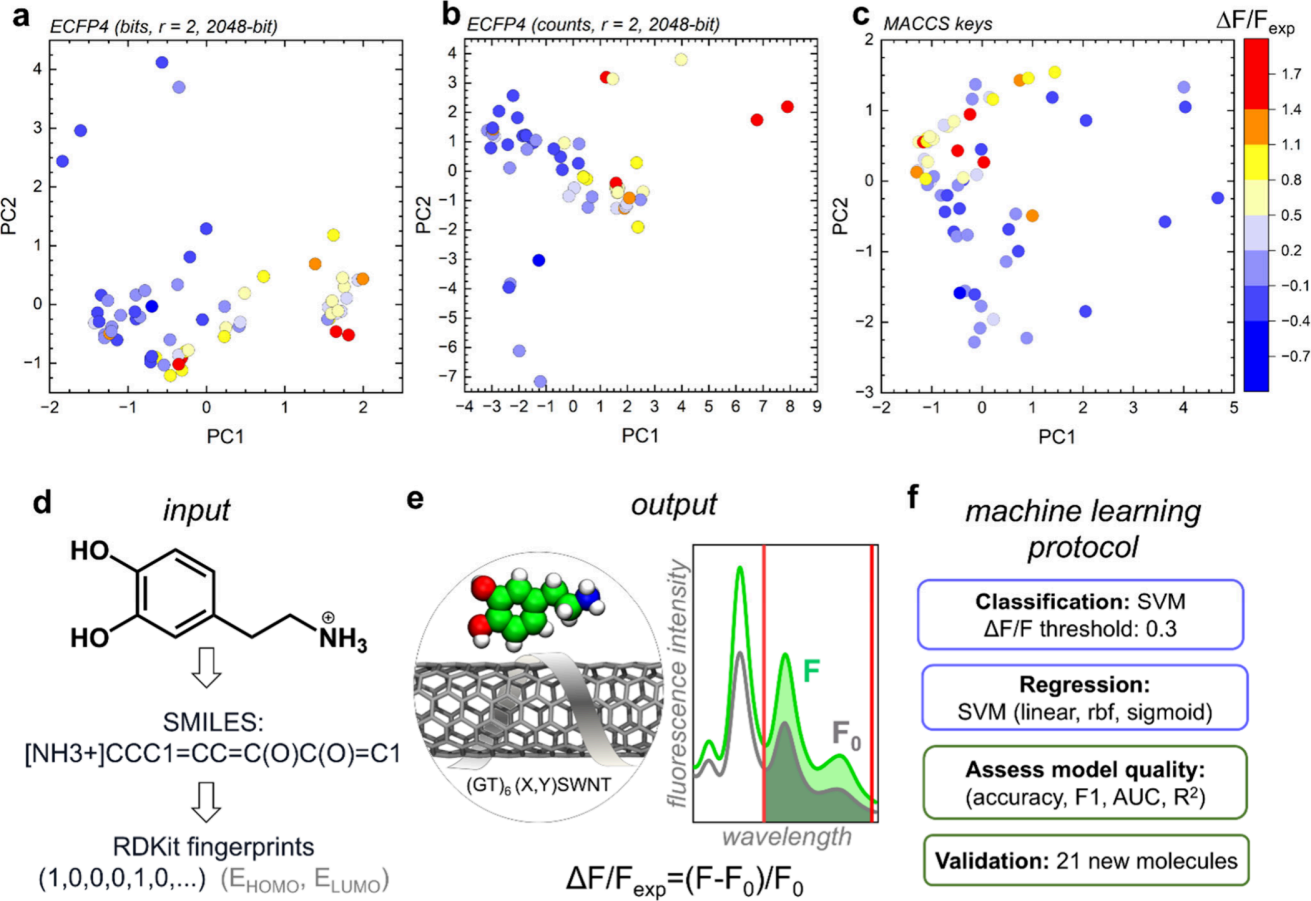
Chemical space covered by the data set and machine learning workflow to predict optical responses of the (GT)_6_ ssDNA–SWCNT nanosensor to molecular analytes. (a) PCA plot for molecules encoded using the ECFP4 bit vectors, with radii of 2 and 2048 dimensions. (b) PCA plot for molecules encoded using the ECFP4 counts vectors, with radii of 2 and 2048 dimensions. (c) PCA plot for molecules encoded using the MACCS keys. (d) Molecules in the data set are encoded as SMILES and converted into molecular fingerprints with RDKit. In some models, HOMO and LUMO energies of molecules are appended as additional descriptors. (e) Models are trained to predict the values of the sensor’s optical response, Δ*F*/*F*_exp_, defined as (*F* – *F*_0_)/*F*_0_, where *F*_0_ and *F* are areas under the fluorescence intensity curves in the wavelength range from 1100 to 1400 nm before and after analyte addition, respectively. (f) Support vector classifiers and regression models are developed. The model performance is tested and assessed on the original data set and on a 21-molecule blind data set not seen during the training.

Since PC1 values for ECFP4 bit vectors significantly correlated with the sensor response, we examined the structural features associated with this component and identified chemotypes and molecular descriptors associated with the largest absolute PC1 coefficients (Tables S2 and S3). Negative PC1 coefficients, enriched with weak-response molecules, corresponded primarily to unsubstituted aromatic carbons and phenoxide motifs. Positive PC1 coefficients, enriched with strong-response molecules, corresponded to phenolic and oxygenated aromatic fragments. Correlations with simple molecular descriptors supported this trend, as PC1 correlated with hydrogen bond donors (ρ = 0.55), topological polar surface area (ρ = 0.36), and oxygen count (ρ = 0.34). Together, these results highlight oxygenated aromatics as a structural motif driving strong nanosensor responses.

To predict continuous Δ*F*/*F* values, we trained SVR and SVC models with multiple fingerprint-kernel combinations. The workflow (Figure 1d–f) involved encoding molecules into RDKit fingerprints,^37^ with or without highest occupied molecular orbital (HOMO) and lowest unoccupied molecular orbital (LUMO) energy descriptors, to train models that either predict continuous Δ*F*/*F*_ML_ values (regression, SVR) or classify responses as high (class 1) or low (class 0) (classification, SVC). Individual models occasionally achieved strong performance (e.g., linear SVR with AtomPairs fingerprints; *R* ^2^ = 0.82; *r*_Pearson_ = 0.91; *p* < 0.0001 (Figure 2a)). However, given the small data set size, model training exhibited stochastic variability. To systematically evaluate the model stability, we used two complementary approaches. First, we trained 200 SVR models for each fingerprint-kernel combination with various random seeds. Performance distributions were broad (Figures S3 and S4), with *R* ^2^ values ranging from negative (as low as −7) to near 0.9. Mean *R* ^2^ values across ensembles ranged from −0.65 to 0.36 (Figure 2b and Figures S3 and S4). Inclusion of HOMO and LUMO descriptors generally did not improve the performance, indicating that molecular fingerprints alone were more effective. These results highlight both the potential and instability of SVR on small data sets. Second, we employed repeated 5 × 5 nested cross-validation, with and without hyperparameter optimization. Cross-validation models with default hyperparameters achieved mean *R* ^2^ values of 0.3–0.4 (Figure 2c and Table S5), outperforming ensemble averages. In contrast, models with tuned hyperparameters afforded slightly lower *R* ^2^ values (0.28–0.33 (Figure S5 and Table S6)). These findings suggest that hyperparameter tuning overfit the limited data set while default hyperparameters yielded more transferable models.

**Figure 2:**
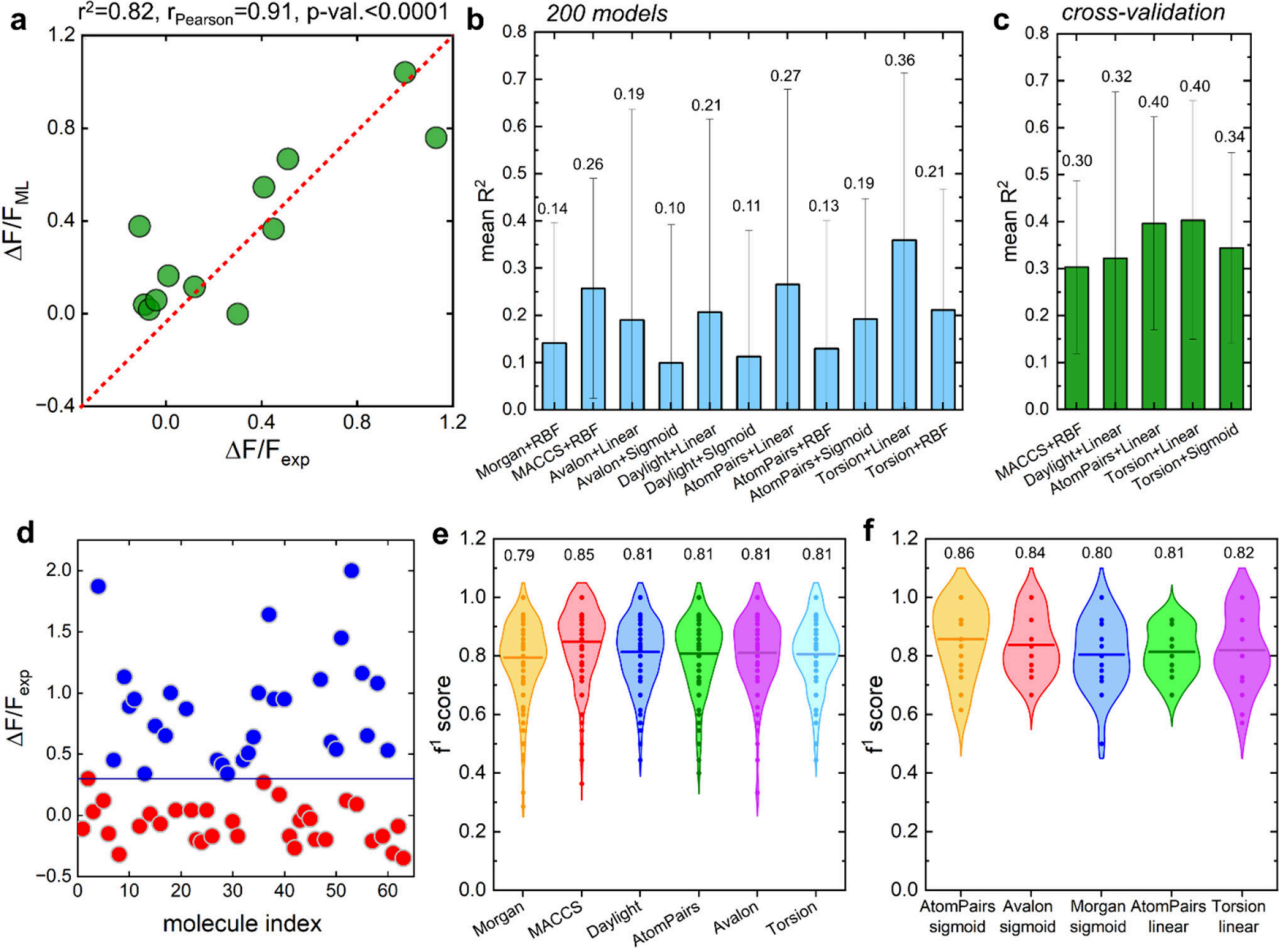
Performance of SVR and SVC models using two approaches for predicting Δ*F*/*F*. (a) Correlation between experimental Δ*F*/*F*_exp_ values and predicted Δ*F*/*F*_ML_ values from one of the best linear SVR models trained with AtomPairs fingerprints (no HOMO or LUMO descriptors). (b) Mean *R* ^2^ values of 200 SVR models for the 11 best-performing fingerprint-kernel combinations. (c) Mean *R* ^2^ values for 5 × 5 cross-validation models with best-performing fingerprints and kernels (Table S5), using default hyperparameters (*C* = 1.0; ε = 0.1; γ = scale for RBF and sigmoid kernels). (d) Optical response Δ*F*/*F*_exp_ to analytes in the initial data set, separated into two classes with a threshold of Δ*F*/*F* = 0.3. (e) Distribution of F1 scores for 200 SVC models using six types of RDKit fingerprints, obtained using different random state variables. (f) Distribution of F1 scores for 5 × 5 cross-validation models. Numbers above the bars or violins in panels b, c, e, and f are mean values of *y*-axis quantities (*R* ^2^, F1 score).

We next simplified the prediction task to binary classification, separating molecules into strong- and weak-response classes using a Δ*F/F* threshold of 0.3 (Figure 2d). This threshold produced an approximately balanced distribution (class 0, 34; class 1, 29; Figure 2d), which is recommended because pronounced class imbalance can hinder classifier learning.^38^ Using this threshold, molecules with negative Δ*F*/*F*_exp_ values were also grouped into the weak-response class for simplicity, with only four of the 63 molecules in our library observed to have negative Δ*F*/*F*_exp_ values of less than −0.3 (Table S1). Ensembles of 200 SVC models achieved mean accuracy and F1 scores of ∼0.8 across fingerprints, with slightly better performance for weak-response predictions (Figure 2e and Figures S6 and S7). Cross-validation SVC models showed similar performance, with mean F1 scores of 0.80–0.86 (Figure 2f and Table S7). As with regression, models trained with default hyperparameters generalized more effectively than tuned models. These results demonstrate that even under data-limited conditions, classifiers robustly distinguish strong- from weak-response analytes.

To evaluate generalizability, we applied an ensemble of our best models, selected from our trained models (listed in Table S4), to a blind set of 21 molecules not included in training (Figure S8 and Table S8). PCA confirmed that these molecules occupied a similar chemical space to the training set (Figure 3a). Regression predictions from 200-model ensembles achieved an *R* ^2^ of 0.264 (Figure 3b). Nested 5 × 5 cross-validation ensembles with default hyperparameters achieved an *R* ^2^ of 0.236, while tuned hyperparameters gave an *R* ^2^ of 0.211 (Figure 3c,d), therefore indicating that tuning for training performance transferred less effectively to blind data. However, because initial testing showed higher *R* ^2^ values (Figure 2c) but blind validation resulted in lower *R* ^2^ values (Figure 3c,d), we also decided to examine the models that use the maximum amount of data per fold, the nested leave-one-out cross-validation approach (LOO-CV). Using Morgan/ECFP4 fingerprints and tuned hyperparameters (*C* = 0.03, and *ε* = 0.05) achieved an *R* ^2^ value of 0.296 (Figure 3e). LOO-CV with default hyperparameters (*C* = 1.0, and *ε* = 0.1) achieved a similar *R* ^2^ value. The best-performing individual model from the nested 5 × 5 CV model (MACCS keys, RBF kernel, default hyperparameters) achieved an *R* ^2^ of 0.423. The other top models from the nested LOO-CV approach are reported in Figure S9. Classification models achieved consistent blind-set performance, maintaining F1 scores around 0.8 (Figure 3f). Together, these results show that default hyperparameter cross-validation models offer the best balance of training performance and transferability.

**Figure 3:**
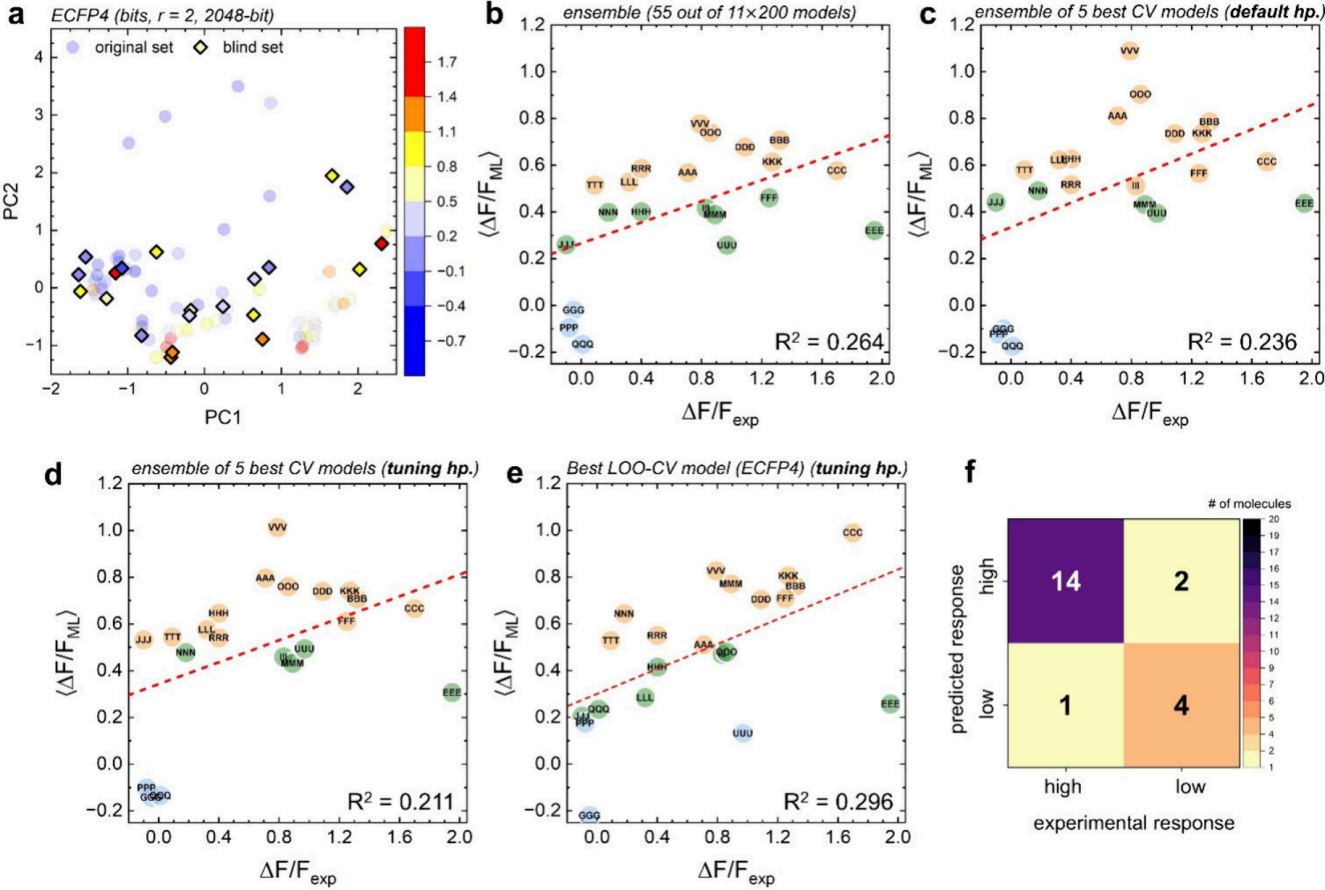
Performance of SVR models in the blind test data set. (a) Chemical space covered by the training set (circles) and blind sets (diamonds) in PCA space. The plot is prepared for molecules encoded with ECFP4 bit vectors (*r* = 2; 2048 dimensions). The color scale shows values of Δ*F*/*F*_exp_, like in Figure 1c. (b) Correlation between experimental Δ*F*/*F*_exp_ and predicted Δ*F*/*F*_ML_ values from an ensemble of the 55 best SVR models, selected from the 200-model approach (from the 11 best fingerprint-kernel combinations). Color code: orange for Δ*F*/*F*_ML_ > 0.5, green for 0.2 < Δ*F*/*F*_ML_ < 0.5, and blue for Δ*F*/*F*_ML_ < 0.2. (c) Correlation from an ensemble of the five best cross-validation models trained on the full data set, with default parameters. (d) Correlation from an ensemble of the five best cross-validation models trained on the full data set, with tuned parameters. (e) Correlation from the best-performing LOO–CV model (highest *R* ^2^ value; tuned hyperparameters, *C* = 0.03 and *ε* = 0.05), trained on the full data set using Morgan/ECFP4 fingerprints and linear kernel. All of the models used for predictions were trained only on RDKit fingerprints without orbital energies. (f) Confusion matrix showing the correlation between our ML-predicted classes (predicted by top 5 classification models trained in a cross-validation approach with hyperparameter tuning) and the Δ*F*/*F*_exp_ responses (using a threshold of 0.3).

This work demonstrates that ML can provide preliminary insight into the chemically meaningful relationships between molecular structure and optical response in DNA–SWCNT nanosensors, despite being trained on modest experimental data sets. By integrating molecular fingerprints, electronic descriptors, and PCA, we identified interpretable structural motifs, such as oxygenated aromatic groups, that are consistently associated with enhanced fluorescence modulation in (GT)_6_–SWCNT dopamine nanosensors. The models reveal that subtle variations in analyte structure, such as the presence of phenolic hydroxyl groups or polar aromatic functionalities, can have a pronounced effect on the sensor response, highlighting the sensitivity of DNA–SWCNT interactions to molecular polarity and hydrogen bonding. These findings are consistent with intuitive expectations based on catechol chemistry, and PCA offers quantitative confirmation of this relationship. Unexpectedly, although redox properties of analytes are known to influence polymer–SWCNT sensing,^39^ adding gas-phase HOMO and LUMO energies reduced model performance, likely because gas-phase orbital energies poorly capture the solution-phase redox potentials and interfacial charge-transfer processes relevant to sensing.

Beyond structural interpretation, our comparative evaluation of SVR and SVC models provides insight into model design for data-limited nanosensor studies. Regression models achieved moderate predictive power (mean *R* ^2^ = 0.2–0.4), while classification models attained strong discrimination between strong- and weak-response analytes (F1 ∼ 0.8). The modest *R* ^2^ values observed for regression are consistent with the sparsity and the limited size of the data set used for model training. These limitations make it difficult for models to learn smooth, continuous structure–response relationships, and they are well-recognized in cheminformatics and materials ML, where small experimental data sets often reduce model stability and resolution.^40,41^ We also found that small-fold CV models with default hyperparameters often generalized better than tuned models. This behavior is typical with limited data, where hyperparameter optimization can overfit to noise in training splits instead of improving true predictive accuracy.^42,43^ In such cases, simpler models often provide more reliable external performance than heavily optimized ones. We therefore view the performance of default models not as an inherent methodological advantage but as a consequence of the data set size. Our framework should be viewed as a conservative, exploratory analysis rather than a high-capacity predictive tool. Finally, we note that our *R* ^2^ values are consistent with those reported in the literature for ML approaches to DNA–SWCNT molecular recognition, underscoring the difficulty of predicting such complex interactions.^29^ While hyperparameter tuning generally reduced transferability in the repeated 5 × 5 cross-validation, the LOO-CV approach showed a different behavior in blind data set tests. In LOO-CV, each training fold contained the maximum amount of data available (62 of the 63 molecules, more than ∼50 molecules in the 5 × 5 cross-validation). With the LOO-CV approach, default and tuned *C* and ε parameters had similar performance using ECFP4 fingerprints for blind test data set. The differences in the repeated K-fold and LOO-CV show the high sensitivity of models to the amounts of data used in small-data regimes. When folds are small, tuning can overfit to noise, whereas when folds are larger, tuning can refine the model capacity in a more controlled manner. However, even with LOO-CV, the gains are moderate, underscoring that the data set size is the primary limiting factor for regression accuracy. Our classification models appear more stable because the classification task is inherently simpler than the regression task. We note that the current framework is an initial, data-limited step toward analyte-centered modeling and not a substitute for larger data sets or atomistic descriptions of nanosensor–analyte interfaces.

We note that the current ML models are likely limited to making predictions within the chemical space spanned by the training data set. Because the analytes were deliberately chosen to sample the region surrounding dopamine, the predictive validity is restricted to this local neighborhood. This limitation is illustrated by a molecule in the blind test set (analyte EEE), for which there is only one similar example in the training data set (RR) and consistently predicted as an outlier (Figure 3b–e), or UUU analyte, which is chemically distinct from the other analytes in the training data set, and is sometimes predicted poorly. Achieving broader generalization will require expanding the training data to include chemically diverse analytes so that the learned structure–response relationships cover a wider applicability domain.

The success of ML-based prediction using only analyte descriptors also highlights a useful advance, that the analyte, not only the DNA corona, could carry predictive information about nanosensor performance. Prior efforts have focused primarily on predicting sequence responsiveness for fixed analytes; in contrast, our analyte-centered approach complements these by mapping how variations in molecular chemistry influence a fixed DNA–SWCNT system. Together, sequence-centric and analyte-centric approaches could provide a foundation for the bidirectional design of SWCNT-based sensors.

Looking forward, expanding training data sets through high-throughput measurements^27^ or incorporating explicit molecular interaction descriptors (e.g., docking energies or molecular dynamics features) could further improve predictive performance and interpretability. Coupling ML with generative or active learning frameworks may also enable iterative, in silico exploration of chemical space to identify optimal analyte–SWCNT pairings with minimal experimental screening.

In summary, this study establishes a framework for predictive analyte-driven modeling of DNA–SWCNT optical responses. By demonstrating that molecular structure encodes sufficient information to forecast sensor behavior, it opens the door to the rational, data-informed design of nanosensors tailored for specific biochemical environments or targets. Such approaches can significantly accelerate the discovery pipeline for next-generation nanomaterial-based sensing platforms. Furthermore, future work could integrate molecular information from high-resolution methods such as molecular dynamics simulations with ML models to explicitly incorporate conformational and steric effects in the DNA corona and to examine whether such insights improve predictive performance. Although demonstrated here for ssDNA–SWCNT species, the analyte-centered ML framework can be applied to other sensors, such as SWCNTs with peptide, polymer, or surfactant coatings, or sensors based on different components, provided that sufficiently large experimental data sets of sensor responses to diverse analytes are available and the analyte chemical structures are known.

## Methods

### Molecular Encoding

The original and blind data sets included 63 and 21 small molecules, respectively, with their Δ*F*/*F*_exp_ responses measured for (GT)_6_–SWCNT species (Figures S1 and S8 and Tables S1 and S8). Δ*F*/*F*_exp_ was defined as (*F* – *F*_0_)/*F*_0_, where *F*_0_ and *F* are fluorescence values before and after analyte addition, respectively. Since Δ*F*/*F*_exp_ values were calculated from the integrated areas under the fluorescence emission spectra (1100–1400 nm), the spectral data were not decomposed into individual chirality contributions and SWCNT handedness was not explicitly considered. To assess peak shifts, we examined spectra for dopamine and several representative compounds (DA, O, RR, T, YY, and Y) and observed only minor changes (0–2 nm) (Figure S2), which were therefore not included in the data sets. Molecules were encoded as SMILES strings and converted into six RDKit fingerprints: RDKit (Daylight-like),^37^ Morgan/ECFP4 (radius 2),^35^ MACCS keys,^36^ Avalon as implemented in RDKit,^37^ AtomPair,^44^ and Topological Torsion.^45^ HOMO and LUMO energies were computed via DFT (Gaussian09, B3LYP/cc-pVTZ) and included as descriptors in some models.

### PCA and Chemotype Analysis

PCA was applied to fingerprint matrices to evaluate the chemical variance. For ECFP4 bit vectors, high-weight PC1 coefficients were mapped back to (SMILES arbitrary target specification) SMARTS substructures and grouped into chemotypes. Label confidence scores and frequency thresholds were used to filter results (Tables S2 and S3). Molecular descriptors, such as topological polar surface area, hydrogen bond donors, and oxygen count, were correlated with PC1 using Spearman coefficients.

## Machine Learning Models

### Two Hundred-Model Ensembles

For each fingerprint-kernel combination, 200 SVR or SVC models were trained with varied random seeds using scikit-learn. Performance distributions were summarized as *R* ^2^ (regression) or F1 scores (classification).

### Cross-Validation

Nested repeated 5 × 5 cross-validation was used for both regression and classification. The outer folds provided unbiased performance estimates, while the inner folds optimized the SVM hyperparameters. For SVR, the grid search varied regularization strength *C* among values of 0.1, 1.0, and 10.0; insensitive-loss parameter ε among values of 0.01, 0.1, and 0.5; and, for nonlinear kernels, radial basis function width γ among values of “scale”, 0.1, and 1.0. For SVC, the tuning grid explored the same set of *C* values (0.1–10.0) and γ values (“scale”, 0.1, and 1.0). Default models corresponded to scikit-learn’s built-in SVM settings-RBF kernel with *C* = 1.0, *ε* = 0.1, and γ = “scale” for SVR and *C* = 1.0 and γ = “scale” for SVC. Stratified folds were used for classification to preserve class balance.

In addition to the repeated 5 × 5 cross-validation model described above, we implemented a nested LOO-CV framework to evaluate whether using the entire data set for training, minus one held-out molecule, would improve model generalization given the small sample size (63 analytes). LOO-CV maximizes the amount of data available to the model in each training fold, which is particularly advantageous in chemical sensing applications with small data sets. The LOO-CV outer loop provided unbiased predictions for every molecule, while an inner repeated 5 × 5 CV simultaneously performed hyperparameter tuning for the SVR models. The default SVR parameters for LOO-CV were a linear kernel with *C* = 1.0 and *ε* = 0.1. During tuning, we explored a grid of regularization strengths *C* ∈ {0.01, 0.03, 0.1, 0.3}­and insensitive-loss parameters ε ∈ {0.02, 0.05, 0.1}, chosen to bias the model toward moderate regularization^46^ and avoid overfitting in the high-dimensional fingerprint space. This nested LOO-CV strategy therefore complemented the repeated K-fold analysis by enabling every data point to contribute to model training, providing a finer-grained assessment of predictive stability across molecules, and identifying optimal fingerprint–hyperparameter combinations under maximal training data availability.

### Evaluation Metrics

Regression models were evaluated using *R* ^2^, while classification models were assessed using accuracy, precision, recall, and F1 scores.

Additional methodological details are provided in the Supporting Information.

## Supplementary Material



## Notes

Detailed methodological information, experimental data set used to train and test the machine learning models, chemotypes and chemical descriptors associated with the first principal component in the PCA, best-performing fingerprint–kernel combinations and hyperparameters for regression and classification models, experimental data set for blind test model validation, structures of molecules in the initial experimental data set, several examples of fluorescence emission spectra before and after the addition of analytes, performance of regression and classification models for different fingerprint–kernel combinations and default or optimized hyperparameters, structures of molecules in the blind test data set, performance of several SVR models for the blind test data set using LOO-CV and 5 × 5 CV, and distributions of HOMO and LUMO energies of molecules in the experimental data sets (PDF)
