# Supplementary material for: Machine Learning Prediction of Analyte-Induced Fluorescence Perturbations in DNA-Functionalized Carbon Nanotubes
Source: Nano Lett. 2026 Jan 7;26(2):787–94. doi: 10.1021/acs.nanolett.5c05206 (PMC12833838; doi:10.1021/acs.nanolett.5c05206)
Supplement: Supplementary file 1 [file nl5c05206_si_001.pdf]

Machine Learning Prediction of Analyte-Induced Fluorescence Perturbations in DNA-Functionalized Carbon Nanotubes

Sayantani Chakraborty,[1+] Andrew T. Krasley,[2+] Colby H. Smith,[1] Abraham G. Beyene,[2*] Lela Vuković[1,3,4*]

[1]Department of Chemistry and Biochemistry, University of Texas at El Paso, El Paso, Texas 79968, United States
[2]Janelia Research Campus, Howard Hughes Medical Institute, Ashburn, Virginia 20147, United States
[3]Computational Science Program, The University of Texas at El Paso, El Paso, Texas 79968, United States
[4]Bioinformatics Program, The University of Texas at El Paso, El Paso, Texas 79968, United States

Email: lvukovic@utep.edu, beyenea@janelia.hhmi.org; [+]authors contributed equally to this work.

Electronic Supporting Information

Detailed Methodological Approaches

Encoding molecular structures. The datasets used in this study comprised 63 original set small molecules (**Figure S1**) and additional 21 blind set molecules (**Figure S7**) and their experimental optical responses, $\Delta F/F_{exp}$ (**Tables S1, S8**) measured after their addition to a solution of $(GT)_6$-DNA-SWCNT conjugates. The data for the initial dataset of 63 molecules was first reported in Ref.[1] while the dataset for 21 molecules is first reported here, and was obtained using the same experimental techniques as described in Ref.[1] The value of $\Delta F/F_{exp}$ is defined as $(F-F_0)/F_0$, where F_0 and F are areas under the fluorescence intensity curves in the wavelength range from 1100 nm to 1400 nm before and after analyte addition, respectively.

Molecular structures were encoded as simplified molecular-input line-entry system (SMILES)[2] which were then converted to six molecular fingerprints with the RDKit (RDKit: Open-source cheminformatics. https://www.rdkit.org) library: RDKit (Daylight-like) fingerprint (minimum-maximum path of 1-7, 2048 bits in bit vectors, no explicit hydrogens, branched paths on, bond order used, chirality ignored); Morgan/ECFP4 (radius 2, 2048 bits in bit vectors, no explicit hydrogens, chirality ignored); MACCS keys (167-bit vector, no explicit hydrogens, chirality ignored); Avalon (1024 bits in bit vectors, no explicit hydrogens, chirality ignored); AtomPair (minimum-maximum topological distance of 1-30 bonds, 2048 bits in bit vectors, chirality ignored, hashed bit vector); and Topological Torsion (torsion length of 4 atoms, 2048 bits in bit vectors, chirality ignored, hashed bit vector).[3–5]

In some models, HOMO (highest occupied molecular orbital) and LUMO (lowest unoccupied molecular orbital) energies of the molecules were appended as additional descriptors (**Table S1, Figure S8**). These orbital energies were obtained from density functional theory (DFT) optimization calculations performed in Gaussian09,[6] using the cc-PVTZ basis set[7,8] and B3LYP functional[9] in the gas phase.

Principal component analysis and interpretation of chemotypes. PCA was performed on molecular fingerprints to examine chemical variance in the dataset. Three representations were tested, namely, ECFP4 (r = 2, 2048-bit vectors) in both bit and count formats, and MACCS keys.

A Python workflow was used to interpret which chemical features span the range of PC1 values for molecules encoded using ECFP4 molecular fingerprints (r = 2, 2048-bits in bit format). This workflow generated annotated fingerprints, applied PCA, and mapped the bits with highest absolute values of positive and negative coefficients contributing to PC1 (c_{PC1}) back to their contributing substructures, expressed as SMARTS patterns and classified into human-readable chemotypes (e.g., aromatic carbon scaffold, protonated amines, phenolic groups). For each bit, the workflow reported its frequence of occurrence in the dataset and a label confidence score (the fraction of all SMARTS substructure occurrences for the selected bit that contribute to the primary chemotype). Furthermore, simple molecular descriptors (TPSA, hydrogen bond counts, logP, molecular weight, aromatic/aliphatic ring counts, heteroatom counts) were calculated, and their correlations with PC1 are quantified using Spearman coefficients.

Machine learning models.

200-models approach. Molecular fingerprints generated as described above were used as input for training regression and classification models. The dataset was split into training (80%, 50 molecules) and testing (20%, 13 molecules) subsets. Regression models were implemented in scikit-learn[5] using support vector regression with linear, sigmoid, or radial basis function kernels.[10]For each fingerprint type, 200 SVR models were trained by varying random state variables to predict $\Delta F/F_{ML}$ values for the test molecules. This approach was based on our work in Refs.[11,12] Classification models were trained using the support vector classification algorithm.[13] Molecules were assigned to binary response classes, with class 0 corresponding to low response and class 1 to high response molecules. Threshold values for separating the two classes were evaluated, and a threshold of 0.3 was selected, since it produced the most balanced

class distribution. For each fingerprint, 200 SVC models were trained by varying random seeds to predict the classes of molecules in the test set.

5 × 5 cross-validation regression with tuned hyperparameters. Separately, we trained SVR regression models using the above-described RDKit fingerprints as input features and a nested cross-validation (CV) protocol. The outer CV loop used repeated K-fold (5 folds × 5 repeats, randomized splits) on the 63 labeled molecules. The inner CV loop used GridSearchCV with 5-fold CV to select the fingerprint type, kernel, and hyperparameters that maximize mean R^2. We evaluated SVR with linear, sigmoid, and RBF kernels, by varying C values ({0.1, 1, 10}) and ε values ({0.01, 0.1, 1.0}) for all kernels. For RBF and sigmoid kernels, we also varied γ values ({scale, 0.01, 0.1}). In each outer-loop split, the inner loop selected the best fingerprint, kernel, and hyperparameter settings. A model with these settings was then trained on the outer training folds and evaluated on the corresponding test fold. Performance for each fingerprint-kernel-hyperparameter setting is reported as the mean ± standard deviation of R^2 across the 25 outer evaluations. Finally, the settings that led to the highest R^2 were used to retrain models on all 63 molecules, which were then applied to blind-set predictions.

5 × 5 cross-validation regression models with default hyperparameters. We also trained SVR regression models using repeated 5-fold CV (5 folds × 5 repeats), and where only the fingerprint and kernel types were varied, while the SVR hyperparameters were kept at their default values (C = 1, ε = 0.1 and γ = scale).

Leave-one-out cross-validation (LOO-CV) regression models. These models are described in the Methods within the main manuscript document.

Cross-validation classification models. Molecules were assigned to binary response classes using a threshold of $\Delta F/F_{exp} \geq 0.3$ (as described above). We trained SVC models in a nested cross-validation scheme. The outer loop used repeated stratified 5-fold CV (5 folds × 5 repeats) to estimate model performance, and the inner loop used stratified 5-fold GridSearchCV to select the kernel and hyperparameters; the stratified approach was used to preserve class ratios. We evaluated SVC models with linear, sigmoid, and RBF kernels, both with default hyperparameters and with hyperparameter tuning (as in the SVR models). For each fingerprint-kernel setting, performance was reported as mean accuracy and f^1 score ± standard deviation across the 25 outer evaluations. Finally, the top-ranked models were trained on all 63 molecules and applied as an ensemble to predict the classes of the blind-set molecules.

All codes used in training ML classification and regression models are available on GitHub (https://github.com/vukoviclab/dna-nanotube-nanosensor-ML).

Evaluation metrics. Regression models were trained to predict ΔF/F values for new molecules. Model performance was evaluated using the coefficient of determination (R^2), where higher values indicate better agreement between predicted and observed test set responses. Classification models were trained to predict the probability of each molecule belonging to either binary response class. Model performance was evaluated using accuracy, precision, recall, and F1 score. Accuracy measures the overall correctness, precision provides the fraction of predicted positives that are true, recall provides the fraction of actual positives correctly identified, and the F1 score balances precision and recall.

Table S1. Dataset of molecules used for training ML models. Molecule IDs refer to molecule structures in Figure S1. The experimental optical responses of $(GT)_6$-SWCNT sensors to molecules, $\Delta F/F_0$, and calculated HOMO and LUMO energies of molecules (DFT optimization, B3LYP/cc-PVTZ in gas phase).

Compound ID	$\Delta F/F_0$	HOMO (eV)	LUMO (eV)
1	-0.11	-6.05154302	-0.230752664
2	0.30	-0.694162788	3.7600711
3	0.03	-5.88337658	-0.671849441
4	1.87	-5.39602042	-0.895799255
5	0.12	-8.3443755	-4.92172574
6	-0.15	-0.840288001	3.0185605
7	0.45	-5.76364642	-0.11592056
8	-0.32	-5.28635848	-0.430756446
9	1.13	-9.48099564	-4.74512376
10	0.89	-5.40064636	-0.339054032
A	0.95	-6.09997931	-0.577698001
AA	-0.09	2.3605889	9.1490166
B	0.34	-1.34043351	3.3410156
BB	0.01	-7.49102603	0.47701582
C	0.73	-5.56364264	-0.239188197
CC	-0.07	-7.0333303	-2.81910094
D	0.65	-5.79112994	-0.179595233
DA	1.00	-9.40507584	-4.6370945
DD	0.04	-6.03521618	-0.191296135
E	-0.67	-6.6910109	-3.43952083
EE	0.87	-5.83303549	0.15020692
F	0.04	-5.23111934	-0.054967026
FF	-0.20	-6.67794943	-2.18861282
G	-0.22	-6.08310825	-0.466947607
GG	0.04	-5.84936233	-0.090341845
H	-0.17	-6.57781148	-0.419871887
HH	0.45	-1.31458269	1.9842552
I	0.41	-6.36529045	-1.5600295
II	0.34	-6.39250185	-1.24546573
J	-0.05	-7.03088127	-1.79840136
JJ	-0.17	-1.33199798	2.9913491
K	0.45	-6.61563532	-1.39839379
KK	0.51	-5.50622659	-0.151023264
L	0.64	-6.43277472	-1.74261799
LL	1.00	-6.27630918	-0.49715226
M	0.27	-5.93208498	-1.15485177
MM	1.64	-5.9421532	0.136057
N	0.95	-6.20392686	-0.173064498
NN	0.17	-6.15385788	0.20680663
O	0.95	-5.97426265	-0.259324632
OO	-0.17	-9.8478053	-4.77750532
P	-0.27	-6.46379572	-0.509669503
PP	-0.04	2.2977305	6.8937358
Q	0.03	-7.06734455	-0.788314229
QQ	-0.03	-8.08124128	-1.05553017
R	-0.20	-0.43102856	4.0476956
RR	1.11	-5.25833074	0.081362083
S	-0.20	-0.15619343	4.0060622
SS	0.60	-5.49942374	-1.49853174
T	0.54	-5.96392232	-0.20599029
TT	1.45	-5.85289981	-0.438103524
U	0.12	-1.8283339	3.2278161
UU	2.00	-1.41825812	1.4030197

V	0.09	-7.13265191	-2.71869087
VV	1.16	-6.04963823	-1.7739111
W	0.65	-5.82106247	-1.0892723
WW	-0.21	-5.9946712	-0.888452177
X	1.08	-1.46968766	3.2721707
XX	-0.17	-5.86296803	-0.296876363
Y	0.53	-5.82650475	-0.200275897
YY	-0.31	-8.68098051	-4.99356383
Z	-0.09	-6.66488796	-1.67975966
ZZ	-0.35	-9.72480977	-4.7285248

Table S2. Chemotypes associated with the first principal component of ECFP4 bit vectors of molecules in the dataset. Rows are ECFP4 bits (2048-bit, radius = 2) whose label confidence is ≥ 0.5 and that satisfy frequency ≥ 7 or $|c_{PC1}|$* frequency ≥ 0.5. Here, c_{PC1} is the bit's coefficient in PC1 and frequency is the number of molecules in the dataset whose fingerprint contains that bit. The direction column in the table reports the sign of c_{PC1}. The label confidence is the fraction of all SMARTS (SMILES ARbitrary Target Specification) substructure occurrences for that bit that vote for the primary chemotype (weighted majority); the obtained SMARTS for top 20 positive and negative bits are reported in a more detailed table (csv file attached as an additional supplementary information).

bit	c_{PC1}	direction	frequency	c_{PC1} *frequency	⟨c_{PC1}⟩ *(if on)*	example SMILES (fragments)	primary label of fragments	label confidence
1088	-0.286	negative	31	8.859	-0.769	C-C=C; C-C(-C)-O	aromatic carbon scaffold	0.982
1199	-0.274	negative	22	6.022	-1.037	C-C=C-C=C	aromatic carbon scaffold	1.000
1349	-0.096	negative	7	0.669	-1.138	C-C(-C)=C; O-C-O	aromatic carbon scaffold	0.875
1831	-0.084	negative	14	1.169	-0.497	C-C(-O)=C-C=C; C-C=C-C(-O)=C	aryl-O (phenolic/phenoxy)	1.000
1	-0.062	negative	7	0.433	-0.737	C; C-C(-S)=C-C=C	misc	0.909
1226	-0.049	negative	7	0.343	-0.584	C-[O-]	carboxylate/phenoxide	1.000
695	0.041	positive	9	0.366	0.377	O; C-C(=C)-C=C(-O)-C; C-C(=C-C(-O)=C)-C; C-C(-O)=C(-O)-C(-O)=C	misc	0.750
1750	0.047	positive	57	2.672	0.069	C-C=C	aromatic carbon scaffold	1.000
745	0.061	positive	9	0.550	0.566	C-C(-O)=C; C-C(-O)=C-C(-O)=C	aryl-O (phenolic/phenoxy)	0.929
1607	0.243	positive	38	9.228	0.533	C-C(-O)=C; C-C(=C(-C=C)-C(-C)=C)-N; C-C=C(-C(-C)=C)-C(=C)-N	aryl-O (phenolic/phenoxy)	0.968
1602	0.272	positive	43	11.683	0.527	C-O; C=C-C(=O)-C(-[O-])=C	misc	0.988
315	0.345	positive	19	6.559	1.515	C-C=C(-O)-C(-O)=C; C-C(-O)=C(-O)-C=C	di-oxygenated aromatic	1.000
1475	0.346	positive	18	6.224	1.602	C-C(-O)=C-C=C; C-C=C-C(-O)=C	aryl-O (phenolic/phenoxy)	1.000
589	0.355	positive	19	6.740	1.557	C-C=C(-O)-C(-O)=C; C-C(-O)=C(-O)-C=C	di-oxygenated aromatic	1.000
875	0.357	positive	25	8.923	1.191	C-C=C	aromatic carbon scaffold	1.000

Table S3. Correlations of RDKit chemical descriptors (rows) with principal component 1 coefficients (c_{PC1}) from the PCA of the initial dataset (molecules represented as ECFP4 bit vectors). Columns show: Spearman correlation coefficient for each descriptor and c_{PC1}, the p-value of the correlation, mean descriptor value for molecules with positive c_{PC1}, mean descriptor value for molecules with negative c_{PC1}. For the descriptors shown in the rows the full forms for the different abbreviations are Hydrogen Bond Donor (HBD), Topological Polar Surface Area (TPSA), number of oxygen atoms (nO), Hydrogen Bond Acceptor (HBA), Molecular Weight (MW), calculated partition coefficient (cLogP), number of aromatic and aliphatic rings (AromRings and AliphRings), and the number of nitrogen atoms (nN).

descriptor	$\rho_{Spearman}$	p	mean *(if PC1 is positive)*	mean *(if PC1 is negative)*
HBD	0.549201348	3.15E-06	2.541666667	1.487179487
TPSA	0.361597731	0.003593	66.32541667	47.79102564
nO	0.344036495	0.005765	3.125	2.153846154
HBA	0.342360657	0.006023	3.25	2.333333333
HeteroAtoms	0.299530091	0.017085	3.458333333	2.743589744
MW	0.238393588	0.0599	163.2986667	141.4168205
cLogP	0.179400399	0.159464	1.017125	0.591794872
AromRings	0.169357174	0.184535	1.25	1
nN	-0.036519805	0.77629	0.291666667	0.384615385
AliphRings	-0.196261753	0.123166	0.041666667	0.128205128

Table S4. Best-performing combinations of fingerprints and kernels as obtained from regression training models (both 200-model approach and repeated fold cross validation approach without hyperparameter tuning).

**200-model approach**		**5-fold cross validation approach**	
Fingerprint Type	*Kernel Type*	*Fingerprint Type*	*Kernel Type*
Morgan	RBF	MACCS	RBF
MACCS	RBF	Daylight	Linear
Avalon	Linear	AtomPairs	Linear
Avalon	Sigmoid	Torsion	Linear
Daylight	Linear	Torsion	Sigmoid
Daylight	Sigmoid		
AtomPairs	Linear		
AtomPairs	RBF		
AtomPairs	Sigmoid		
Torsion	Linear		
Torsion	RBF		

Table S5. Best performing combination of fingerprints and kernels from classification models trained with 5-fold repeated cross validation without hyperparameter tuning.

**Fingerprint**	**Kernel**
AtomPairs	Sigmoid
Avalon	Sigmoid
Morgan	Sigmoid
AtomPairs	Linear
Torsion	Linear

Table S6. Best performing combination of fingerprints and kernels and their corresponding hyperparameters from regression models trained with 5-fold repeated cross validation with hyperparameter tuning.

Fingerprint	Kernel	C	ε	γ
Torsion	Linear	0.1	0.1	N/A
Daylight	Linear	0.1	0.1	N/A
AtomPairs	Linear	0.1	0.1	N/A
Torsion	Sigmoid	1	0.01	Scale
Torsion	RBF	10	0.01	Scale

Table S7. Best performing combination of fingerprints and kernels and their corresponding hyperparameters from classification models trained with 5-fold repeated cross validation with hyperparameter tuning.

Fingerprint	Kernel	C	γ
Avalon	Linear	0.1	N/A
AtomPairs	Linear	0.1	N/A
Torsion	Linear	0.1	N/A
MACCS	Linear	0.1	N/A
Daylight	Linear	0.1	N/A

Table S8. Dataset comprising of experimentally obtained optical responses ($\Delta F/F_{exp}$) (in 1X PBS solution) and values of HOMO and LUMO energies obtained in DFT optimization calculations using cc-PVTZ basis set and B3LYP functional for 21 molecules used in the blind test to examine the performance of our ML model.

Compound Name	$\Delta F/F_{exp}$	HOMO (eV)	LUMO (eV)
AAA	0.71	-5.79929	0.060681
BBB	1.32	-5.76637	-0.86832
CCC	1.70	-8.33512	-5.12581
DDD	1.09	-9.63855	-4.94594
EEE	1.95	-5.33343	-0.04054
FFF	1.25	-5.89644	-0.12
GGG	-0.05	-6.4638	-0.50967
HHH	0.40	-5.80011	-0.04272
III	0.83	-5.29779	-0.14912
JJJ	-0.10	4.585121	9.396912
KKK	1.27	-5.87412	-0.05714
LLL	0.32	-1.32764	1.17907
MMM	0.89	-5.07955	-3.07489
NNN	0.18	-8.73295	-5.06785
OOO	0.86	-5.29779	-0.14912
PPP	-0.08	-0.20871	3.797079
QQQ	0.01	-1.42452	-6.22923
RRR	0.40	-5.23493	-1.49989
TTT	0.09	-5.32908	-1.37989
UUU	0.97	-0.80192	3.196523
VVV	0.79	-5.1503	0.367082

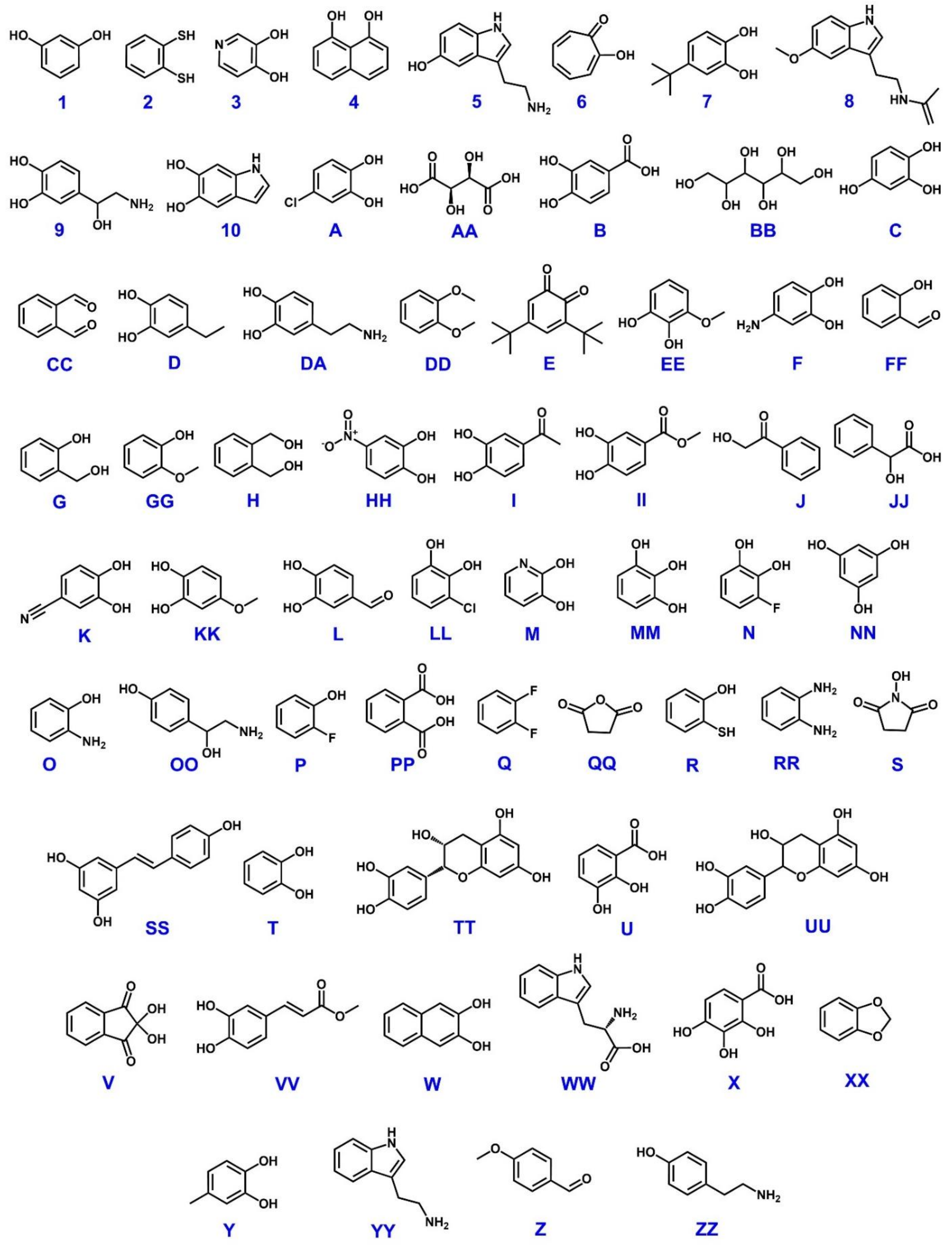

Figure S1. Library of 63 molecules in the initial experimental dataset. Molecules 5, 9, DA, OO, WW, YY, ZZ have positively charged ammonium groups (-NH_3^+), and molecules 6, AA, B, HH, JJ, PP, S, U, UU, WW, and X have deprotonated and negatively charged oxygen or sulfur atoms in SMILES and fingerprints used to train the ML models, based on the pH range (7.41-7.65) in which $\Delta F/F$ values were measured.

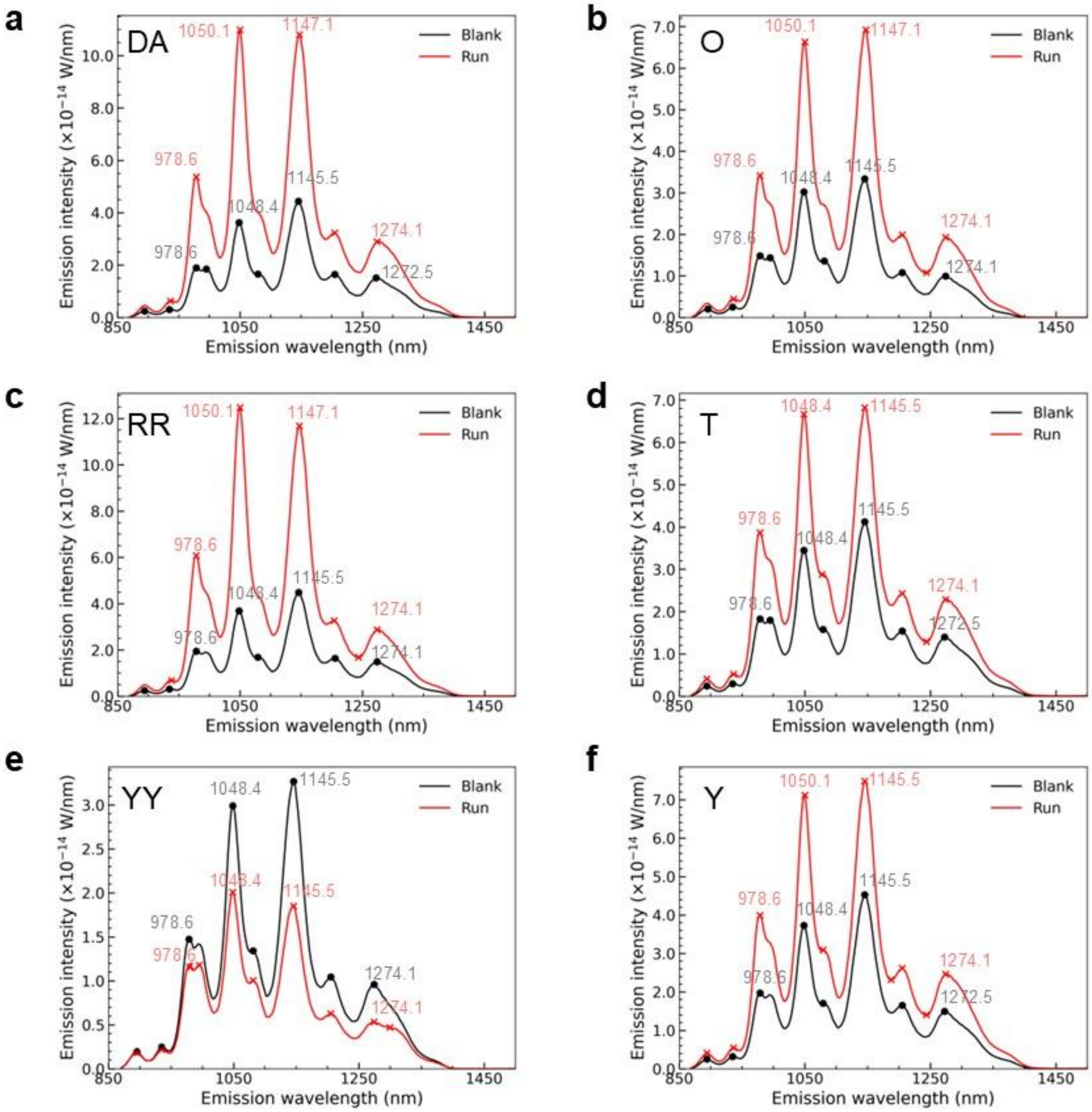

Figure S2. Fluorescence emission spectra of the $(GT)_6$-SWCNT nanosensor solution, prepared from mixed-chirality SWCNT samples, before and after the addition of several analytes. The numbers refer to the wavelengths (in units of nm) observed at maxima of several peaks before and after the addition of analytes. The labels in the plots (DA, O, RR, T, YY, Y) refer to analytes whose structures are shown in Figure S1.

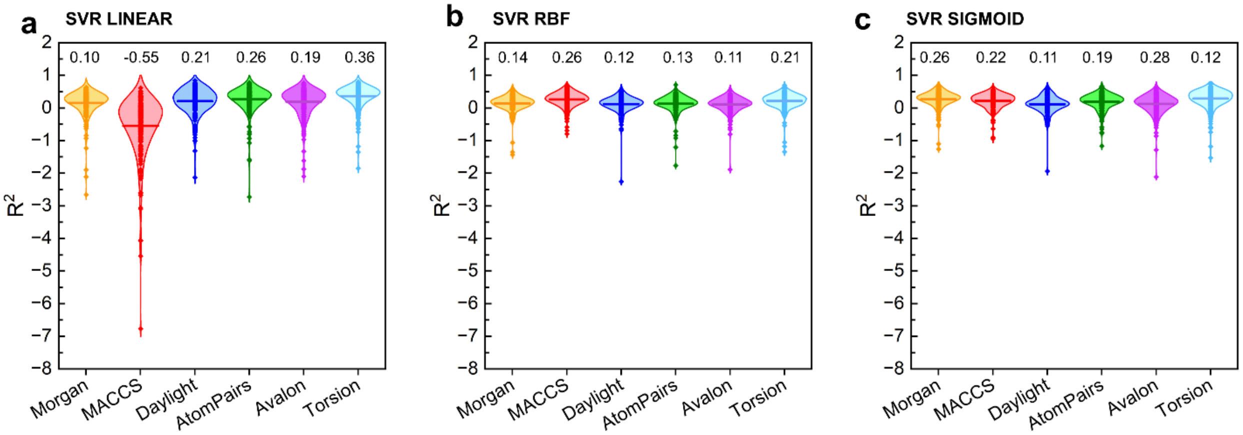

Figure S3. Performance evaluation of the SVR models for different fingerprint-kernel combinations. Violin plot distributions of R^2 values for the six fingerprint types; the molecules are represented as RDKit fingerprints only, using the settings as described in the Methods section.

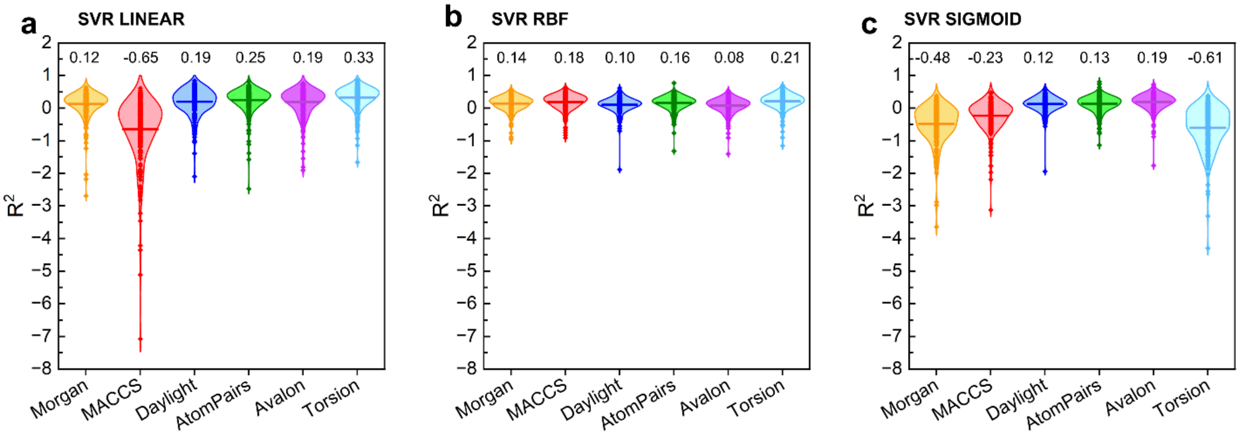

Figure S4. Performance evaluation of the SVR models for different fingerprint-kernel combinations, with the additional HOMO/LUMO energy descriptors. Violin plot distributions of R^2 values for the six fingerprint types; the molecules are represented as RDKit fingerprints with additional HOMO and LUMO energy values added as additional descriptors. The settings used for fingerprints are described in the Methods section.

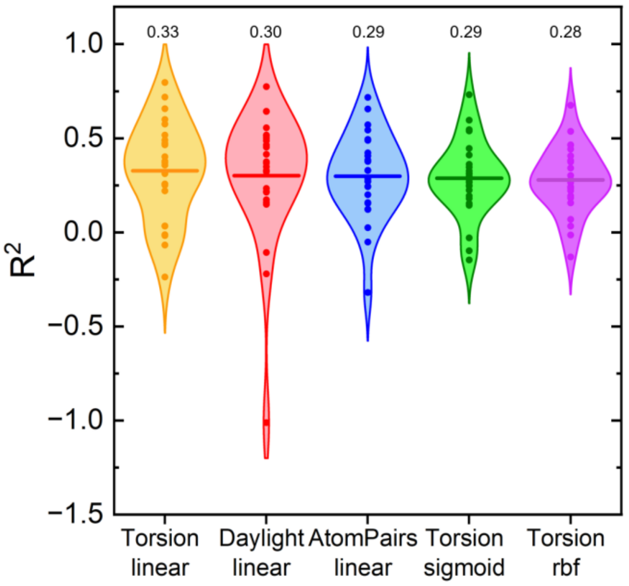

Figure S5. Mean R^2 values for 5×5 cross-validation models with the best fingerprint and kernel combinations, with tuned hyperparameters. Mean values are shown above the bars. All the molecules are represented as RDKit fingerprints (no HOMO/LUMO descriptors).

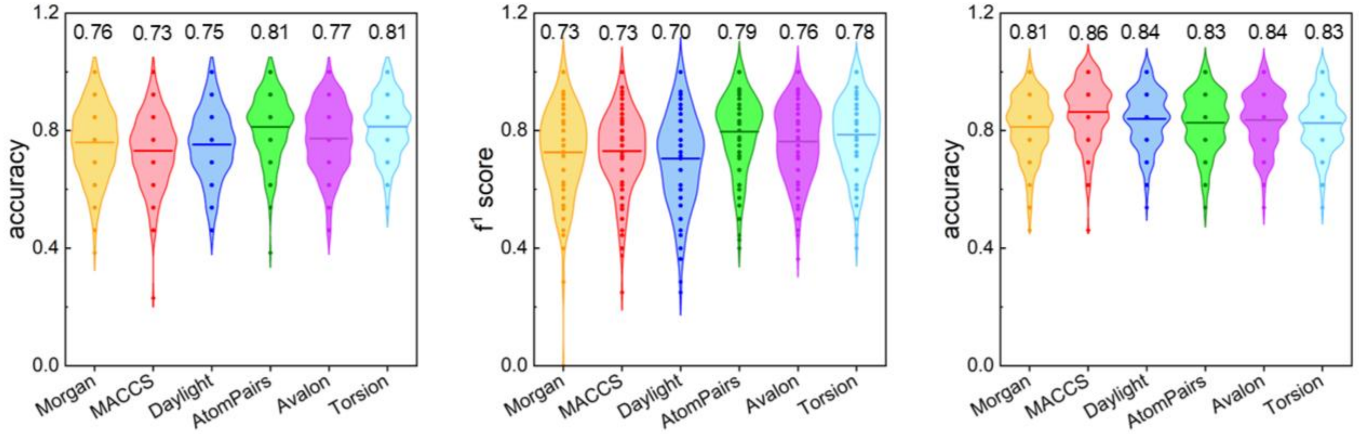

Figure S6. (left, middle) Distribution of accuracy and f^1 score values for SVC models trained with molecular fingerprints and orbital energies as descriptors. (right) Distribution of accuracy values for SVC models trained with molecular fingerprints only.

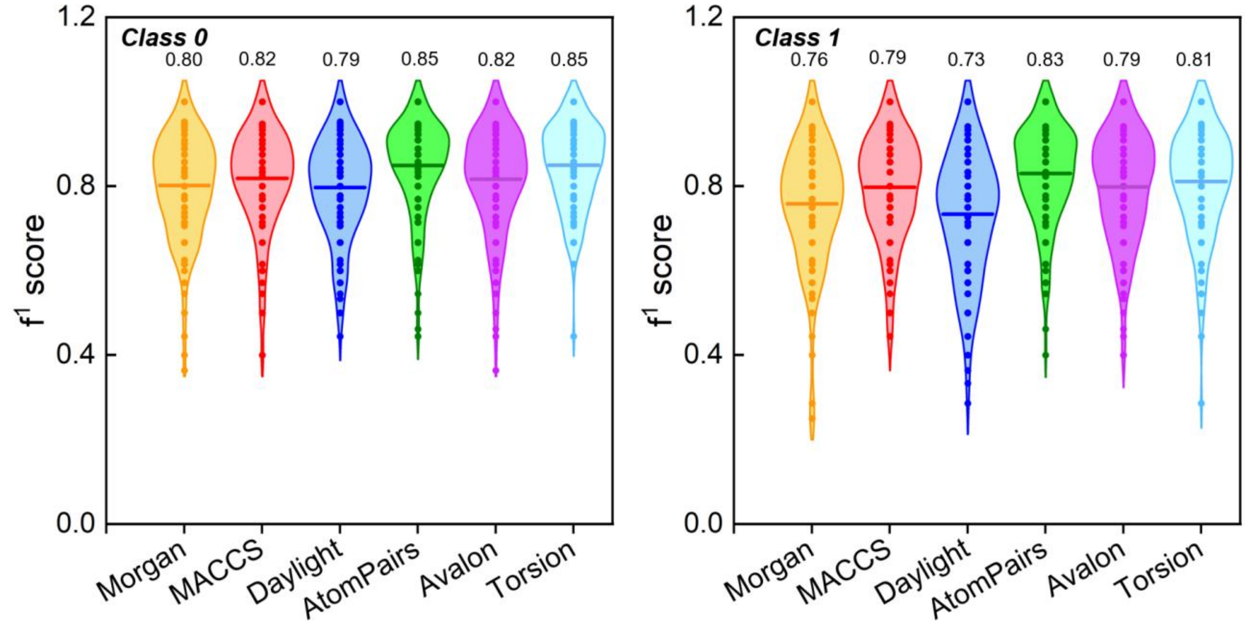

Figure S7. Evaluation of the performance of 200 SVC models per fingerprint type for separate classes. (left) Distributions of F1 scores for predicting molecules that induce low response (class 0). (right) Distributions of F1 scores for predicting molecules that induce high response (class 1).

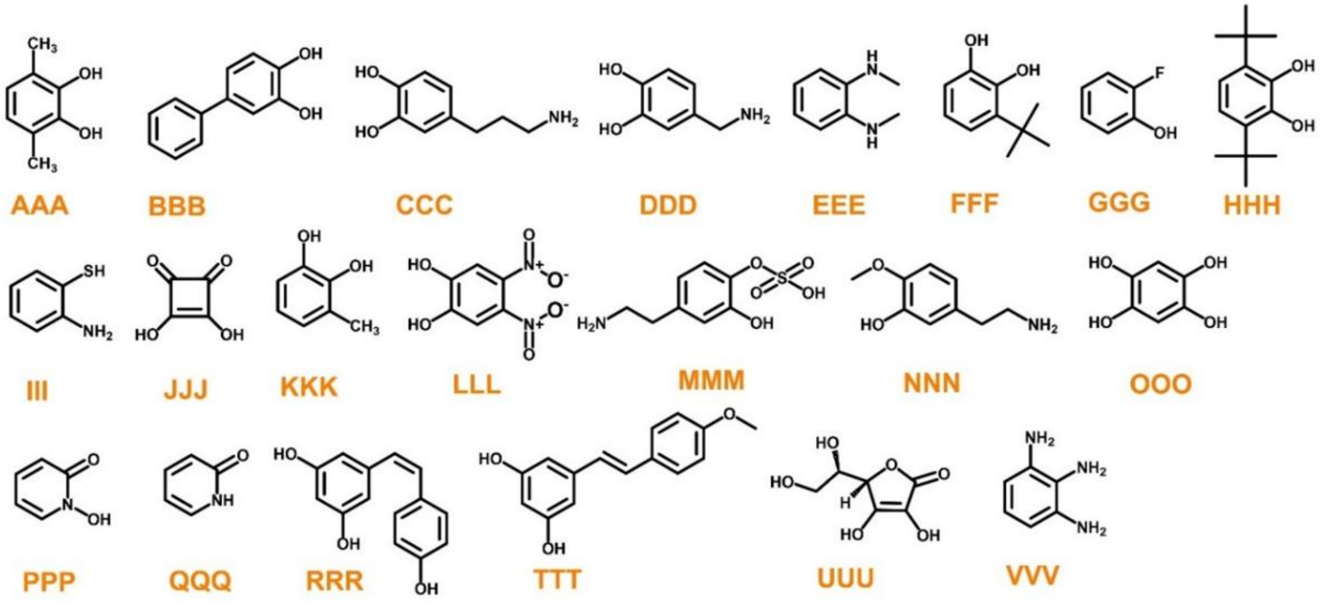

Figure S8. Performance of SVR models in the blind test dataset. a) Library of 21 molecules in the blind test dataset. Molecules CCC, DDD, MMM, NNN have positively charged ammonium groups in SMILES and fingerprints used for making predictions with the ML models. Molecules III, JJJ, LLL, MMM, and PPP have deprotonated and negatively charged oxygen or sulfur atoms in SMILES and fingerprints used for making predictions with the ML models.

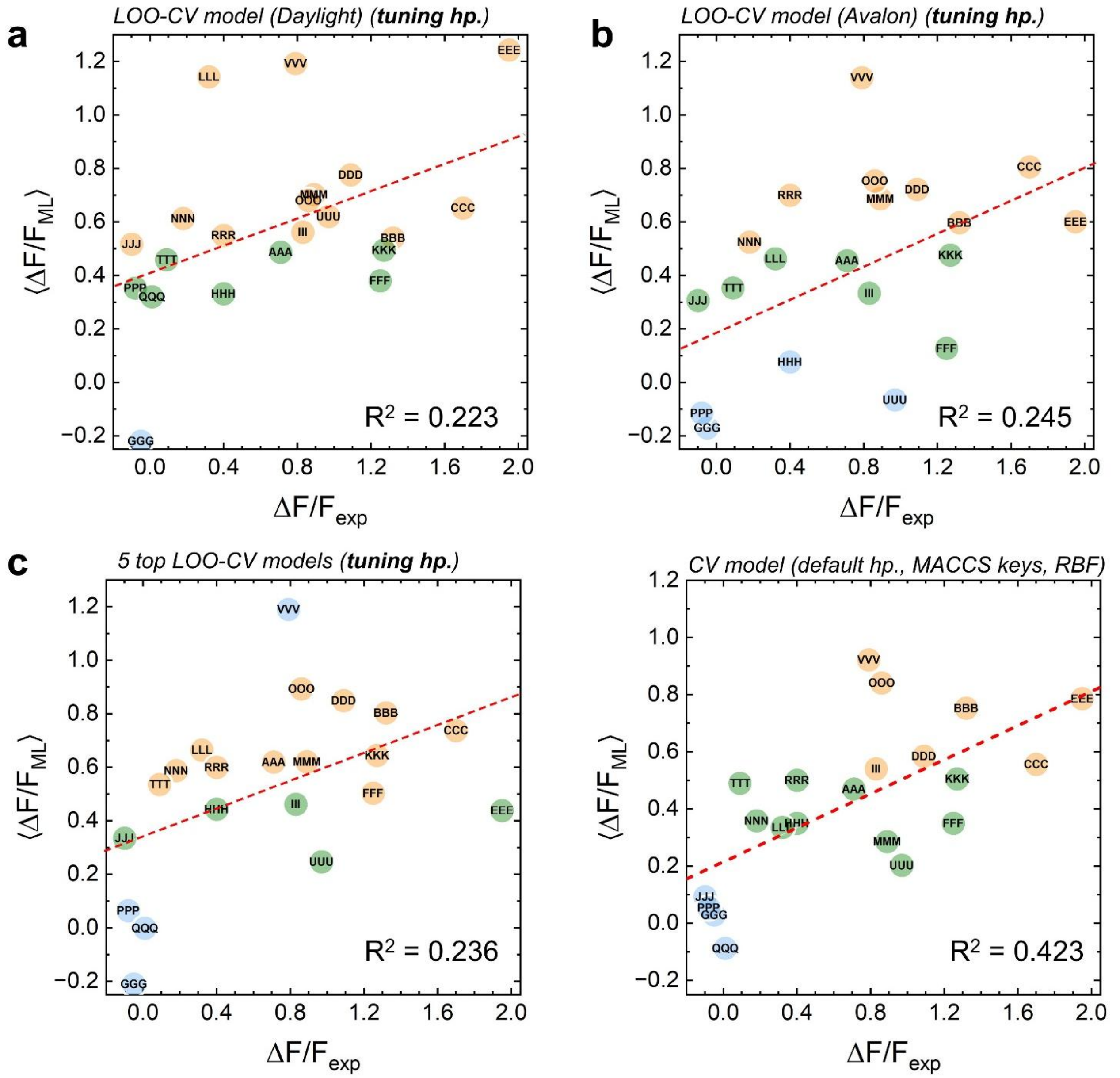

Figure S9. Performance of SVR models in the blind test dataset. a) Correlation between experimental $\Delta F/F_{exp}$ and predicted $\Delta F/F_{ML}$ values from an LOO-CV model trained with Daylight fingerprints, C=0.01 and ε=0.05. Color code: orange: $\Delta F/F_{ML} > 0.5$, green: $0.2 < \Delta F/F_{ML} < 0.5$, blue: $\Delta F/F_{ML} < 0.2$. b) Correlation from an LOO-CV model trained with Avalon fingerprints, C=0.03 and ε=0.1. c) Correlation from an ensemble of the five best LOO-CV models trained on the full dataset, with tuned parameters. d) Correlation from the best-performing 5×5 cross validation model trained with MACCS keys fingerprints and default hyperparameters.

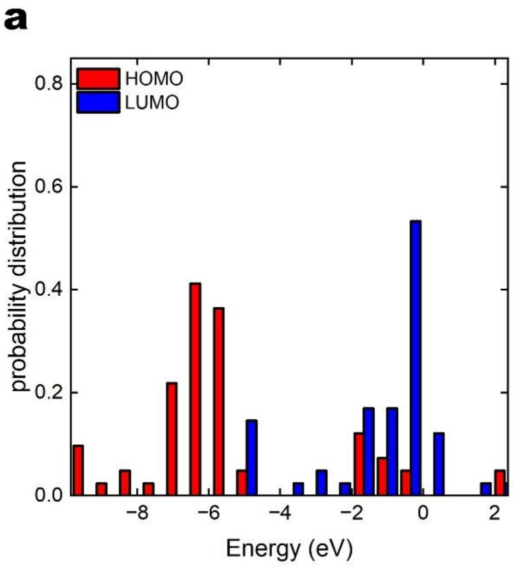

Figure S10. Distribution of HOMO and LUMO energies for a) the original training dataset and b) the new dataset for the blind test.

(1) Krasley, A. T.; Chakraborty, S.; Vuković, L.; Beyene, A. G. Molecular Determinants of Optical Modulation in SsDNA–Carbon Nanotube Biosensors. *ACS Nano* 2025, *19* (8), 7804–7820. https://doi.org/10.1021/acsnano.4c13814.

(2) Weininger, D. SMILES, a Chemical Language and Information System. 1. Introduction to Methodology and Encoding Rules. *J Chem Inf Comput Sci* 1988, *28* (1), 31–36. https://doi.org/10.1021/ci00057a005.

(3) Seo, M.; Shin, H. K.; Myung, Y.; Hwang, S.; No, K. T. Development of Natural Compound Molecular Fingerprint (NC-MFP) with the Dictionary of Natural Products (DNP) for Natural Product-Based Drug Development. *J Cheminform* 2020, *12* (1). https://doi.org/10.1186/s13321-020-0410-3.

(4) Zagidullin, B.; Wang, Z.; Guan, Y.; Pitkänen, E.; Tang, J. Comparative Analysis of Molecular Fingerprints in Prediction of Drug Combination Effects. *Brief Bioinform* 2021, *22* (6). https://doi.org/10.1093/bib/bbab291.

(5) Pedregosa, F.; Varoquaux, G.; Gramfort, A.; Thirion, B.; Grisel, O.; Dubourg, V.; Passos, A.; Brucher, M.; Perrot, M.; Édouardand, M.; Duchesnay, E. Scikit-Learn: Machine Learning in Python. *J Mach Learn Res* 2011, *12* (85). http://jmlr.org/papers/v12/pedregosa11a.html.

(6) Frisch, M. J.; Trucks, G. W.; Schlegel, H. B.; Scuseria, G. E.; Robb, M. A.; Cheeseman, J. R.; Scalmani, G.; Barone, V.; Mennucci, B.; Petersson, G. A.; Nakatsuji, H.; Caricato, M.; Li, X.; Hratchian, H. P.; Izmaylov, A. F.; Bloino, J.; Zheng, G.; Sonnenberg, J. L.; Hada, M.; Ehara, M.; Toyota, K.; Fukuda, R.; Hasegawa, J.; Ishida, M.; Nakajima, T.; Honda, Y.; Kitao, O.; Nakai, H.; Vreven, T.; Montgomery Jr., J. A.; Peralta, J. E.; Ogliaro, F.; Bearpark, M.; Heyd, J. J.; Brothers, E.; Kudin, K. N.; Staroverov, V. N.; Kobayashi, R.; Normand, J.; Raghavachari, K.; Rendell, A.; Burant, J. C.; Iyengar, S. S.; Tomasi, J.; Cossi, M.; Rega, N.; Millam, J. M.; Klene, M.; Knox, J. E.; Cross, J. B.; Bakken, V.; Adamo, C.; Jaramillo, J.; Gomperts, R.; Stratmann, R. E.; Yazyev, O.; Austin, A. J.; Cammi, R.; Pomelli, C.; Ochterski, J. W.; Martin, R. L.; Morokuma, K.; Zakrzewski, V. G.; Voth, G. A.; Salvador, P.; Dannenberg, J. J.; Dapprich, S.; Daniels, A. D.; Farkas, Ö.; Foresman, J. B.; Ortiz, J. V; Cioslowski, J.; Fox, D. J. Gaussian~09 Revision E.01.

(7) Peterson, K. A.; Woon, D. E.; Dunning, T. H. Benchmark Calculations with Correlated Molecular Wave Functions. IV. The Classical Barrier Height of the H+H2→H2+H Reaction. *J Chem Phys* 1994, *100* (10), 7410–7415. https://doi.org/10.1063/1.466884.

(8) Davidson, E. R. Comment on Dunning's Correlation- Consistent Basis Sets. *Chem Phys Lett* 1996, *260*, 514-518.

(9) Tirado-Rives, J.; Jorgensen, W. L. Performance of B3LYP Density Functional Methods for a Large Set of Organic Molecules. *J Chem Theory Comput* 2008, *4* (2), 297–306. https://doi.org/10.1021/ct700248k.
(10) Rodríguez-Pérez, R.; Bajorath, J. Evolution of Support Vector Machine and Regression Modeling in Chemoinformatics and Drug Discovery. *J Comput Aided Mol Des* 2022, *36* (5), 355–362. https://doi.org/10.1007/s10822-022-00442-9.
(11) Kelich, P.; Jeong, S.; Navarro, N.; Adams, J.; Sun, X.; Zhao, H.; P. Landry, M.; Vuković, L. Discovery of DNA–Carbon Nanotube Sensors for Serotonin with Machine Learning and Near-Infrared Fluorescence Spectroscopy. *ACS Nano* 2022, *16* (1), 736–745. https://doi.org/10.1021/acsnano.1c08271.
(12) Kelich, P.; Adams, J.; Jeong, S.; Navarro, N.; Landry, M. P.; Vuković, L. Predicting Serotonin Detection with DNA-Carbon Nanotube Sensors across Multiple Spectral Wavelengths. *J Chem Inf Model* 2024, *64* (10), 3992-4001. https://doi.org/10.1021/acs.jcim.4c00021.
(13) Xue, Y.; Yang, X. G.; Chen, D.; Wang, M.; Chen, Y. Z. Prediction of Antibacterial Compounds by Machine Learning Approaches. *J Comput Chem* 2009, *30* (8), 1202–1211. https://doi.org/10.1002/jcc.21148.
